# Buffalo Academy of Medicine

**Published:** 1900-04

**Authors:** Frederick H. Millener


### Society Proceedings.

### BUFFALO ACADEMY OF MEDICINE.

*Section on Pathology, Tuesday, January 16, 1900.*

Reported by FREDERICK H. MILLENER, M. D., Secretary.

THE fifth regular meeting of the Pathological Section for the season was held on Tuesday, January 16, 1900.

The meeting was called to order by the chairman, Dr. Bennett at 8.50 p.m. Thirty-three Fellows were present.

The minutes of the preceding meeting were read and approved.

The paper of the evening, entitled, "LAWSON TAIT," was read by Dr. STEPHEN Y. HOWELL.

Dr. Howell stated that Lawson Tait became a L.R.C.P. in 1867, and July 29, 1868, he did his first ovariotomy. He was then twenty-four years old. He did five other ovariotomies between that and 1870, when he removed to a suburb of Birmingham. Thus by the time he was twenty-five years old he had done six ovariotomies. In his twenty-fifth year he took his M.R.C.S. and his F.R.C.S., and completed his fellowship in the Royal College of Surgeons, at Edinburgh. Thus early he evinced a want of respect for age, which made him many enemies, as he considered his own opinion equal to that of any one. In 1890, Mr. Tait reached the zenith of his career. For six months in 1892, Dr. Howell had been Mr. Tait's pupil, hence his opportunity to learn much of his character.

In 1893 symptoms of renal trouble supervened, and the last five years of his life were marked with more or less continued invalidism. He was operated on in London, and subsequently developed a chronic nephritis. He was seized on June 3d with renal complications from which he succumbed June 13, 1899.

Dr. WILLIAM WARREN POTTER said that it seemed to him especially fitting that a pause in the busy routine of medical life, such as this, should be made to honor the memory of a deceased member of the profession. Too little attention is given to keeping green the memory of distinguished men; they rise, poise, fall and are gone. The medical men who are thus distinguished are too few. Mr. Tait was one of that number, and he will be long remembered. It is especially appropriate, too, that this section (pathology) should listen to this memorial address. Tait was not a pathologist it is true, but he demonstrated pathology at the operating table. From his experience in operating he established the pathology of extrauterine pregnancy on a firmer basis than it had ever been before.

Dr. Howell has well spoken of the social side of Mr. Tait's life, and the delightful aspects in which it should be reviewed. He was a well-informed man, both in his own work and in the classics and, besides, he was a royal entertainer. He was remarkable in the manner in which he prepared his contributions to the periodical press; everything he ever wrote, whether in local or foreign journals, was carefully sought out and brought to his notice. So, too with every reference made to him in the journals. In earlier days his contributions were dictated but later were written by his own hand. His interest in public affairs was great and his advice was much sought.

He was an intimate acquaintance of Joseph Chamberlain and together they wrought many municipal reforms in the City of Birmingham. The public library in that city is a model and owes much to Mr. Tait.

In looking over his eventful career it seems remarkable that he should find fame in a provincial city, sufficient to bring him patients from all corners of the earth. This fact undoubtedly created jealousy which, together with his rapid rise, led some of his rivals to challenge the correctness of his statistics. His faults made his virtues stand out. No man could attain the notoriety Mr. Tait did without creating many enemies. He was easily irritated. Mr. Tait was very resourceful and few surgeons encountered and surmounted more difficulties than he. One can hardly think of Mr. Tait's career without recalling that of Gregg Smith, whose history in surgical work was similar. Both rose to fame in the provinces, both died young, or at least comparatively so, and each left an enduring fame.

Dr. Matthew D. Mann said he came to listen. He never saw Lawson Tait, but corresponded with him. Dr. Mann stated that Mr. Tait had undoubtedly done considerable for gynecology, but did not originate operations. He resurrected old operations and placed them on a firmer basis. We certainly owe a decrease in the death-rate of many procedures to Mr. Tait's work, and it seemed a pity that he was not more evenly balanced.

Dr. A. G. Bennett stated that he was probably the only physician present who had been a patient of Lawson Tait's. In his younger years Mr. Tait attended him when sick with the measles.

---

*Section on Surgery, Tuesday, February 6, 1900.*

Reported by MARSHALL CLINTON, M. D., Secretary.

THE meeting was called to order by the chairman, Dr. J. Henry Dowd, at 8.45 p.m.

The minutes for the previous meeting were approved.

The essayist for the evening, Dr. Frederic S. Dennis, of New York, was introduced by Dr. Roswell Park. Dr. Dennis at the outset of his remarks paid tribute to the advancements in medical science in which Buffalo has taken an active part, mentioning the cancer laboratory, the introduction of the wet pack in pneumonia, early vivisection by Dalton, and the work in the Health Department on the tubercle bacillus. The first topic of his discourse was:

TUBERCULAR PERITONITIS.

This disease, he said, has only recently been transferred from the realm of medicine to surgery, where it belongs. A positive diagnosis can only be made in this affection by an exploratory laparatomy, and this is always justifiable in cases where there is an ascites that is not due to liver, heart, or kidney lesions. It may be caused by miliary tuberculosis, infected lymph nodes in the mesentery, producing a chronic inflammation and a mass of infected tissues.

Its entrance into the body is by various routes. It may be from the blood, an ulcer of the stomach or intestine which is tubercular, from the ovaries or tubes, or from the genito-urinary tract in the male. From 40 to 50 per cent. are due to a communicating affection from the thoracic lymph nodes and 16 per cent. of all cases are due to infection from the alimentary tract. It may arise as a secondary infection from an infected alimentary tract, as *e. g.*, a tuberculous subject swallowing the tubercle bacillus. Klebs has demonstrated this experimentally by feeding animals pure cultures of the tubercle bacillus. As to age, from Osler's statistics, based on 350 cases we find 27 under 10 years; 75 from 10 to 20 years; 87 from 20 to 30 years; 71 from 30 to 40 years; 61 from 40 to 50 years; 19 from 50 to 60 years; 4 from 60 to 70 years, and 2 from 70 to 80 years. It is more often seen in negroes than in white people and more often in females than in males.

*Symptoms.*—The onset is gradual with a slow gathering ascites. Pressure symptoms from the mass, nausea, vomiting, constipation, rapid pulse, are among the prominent symptoms. The large majority of cases is not diagnosticated before an operation. Osler reports thirty cases diagnosticated before laparatomy, as due to ovarian disease. We may feel nodules due to a puckering together of the omentum, matting together of the intestines or nodes in the mesentery. Pleurisy with bloody effusion and chronic peritonitis means, trauma being excluded, tubercular infection. Beck lays stress on a difference of 2° F. between the rectal and axiliary temperature, as being characteristic of peritonitis with pus. The tumor formed by intestines or omentum may cause pressure symptoms giving rise to jaundice, enlarged superficial veins and the like.

The proper mode of diagnosis without operation is by the systematic use of tuberculin. Give 7-8 mgm. daily, for seven days, and if you get the characteristic reaction rest for a week, then try again; this will give a positive reaction, indicating whether it is a tubercular infection or not. It must be differentiated from subphrenic abscess, typhoid fever, etc.

*Prognosis.*—Age is a great factor in the prognosis of these affections and we find that the middle-aged do better than the young or older people. When there is not much matting together and there is a free exudate, the prognosis is better than when there is no exudate and the tissues are bound down to each other.

The cure of this affection is by laparatomy but the reasons for this are in some dispute. It is probably due to the entrance with the air of nonpathogenic organisms, the stimulus to the peritoneum by these, or to the action of the sunlight which gains access to the areas during the operation. Koch's experiments with direct sunlight would tend to prove the latter. In this disease, as in some others, Americans are the pioneers. Dr. Van de Warker, of Syracuse, in 1883, was the first to report a case successfully treated by simple laparatomy and closure.

The technique has gradually improved until now the ideal operation is to open the abdomen, wash out carefully, empty all pockets where found and close. When purulent, wash and drain all encysted pockets. The method of tapping, inflating with sterilised air, and drainage by iodoform gauze should be done only in pelvic cases. 30 per cent. of all cases are permanently cured and the operation itself has a mortality of only 3 per cent. Fibrous forms may recover without operation, but most of them require it, and it rescues the patient. Do it early for it is never contraindicated even in slight invasions. It is only contraindicated in acute miliary tuberculosis, and when effusion follows the first operation.

Dr. Dennis then presented a case of double tubercular pleurisy with pus in one chest and tubercular peritonitis. Both chests were opened, and the peritoneum opened, cleaned out, and closed. The patient made a phenomenal recovery and the result should stimulate all to operative intervention in these desperate cases.

### GASTROSTOMY.

The surgery of the stomach for the years previous to the last twenty-five has shown a mortality of 82 per cent. Miculicz has lately presented a series of ten cases without a death. The various operations have undergone evolution until the ideal method has been reached. The key-note to this is in the treatment of the stomach wound. An incision five to eight inches long should be made in the stomach wall and through it to the mucous layer and the opening into the stomach should be made at the end of the incision. A rubber tube is now sewn in the incision, forming a gutter, and the stomach sewed to the belly wall. This gutter does not allow regurgitation and subsequent excoriation and discomfort to the patient. This is the ideal operation, as there is no leakage and no mortality.

A patient was then presented that showed the utility of this operation. Stricture of the esophagus followed typhoid fever in July, 1897. In 1898 the patient consulted Dr. Dennis after three months' treatment by gradual dilatation and no relief in a Philadelphia Hospital. On entering the hospital he weighed less than a 100 pounds. His temperature was 96° F. and his pulse 48; he was cyanotic, and in great distress. For three months he had had no food by mouth. Gastrostomy was performed, and while still under the anesthetic, milk and egg were injected into the stomach. The patient began to improve at once, and now after two years weighs 180 lbs., does heavy work, has no leakage and uses alcoholic drinks, which were smelt in the patient's breath. This patient demonstrated the method of feeding himself, after which there was no leakage when coughing with a full stomach. Dr. Dennis's assistant, who accompanied him, reported the patient as having been under the influence of whiskey the day before he was brought to Buffalo for demonstration.

ACUTE APPENDICITIS.

The topic of acute gangrenous appendicitis was next taken up, and it was advised in these cases to let the appendix alone when there was a pus formation and not to handle the gut. The appendix can be removed at a future operation. This is essentially a surgical disease and the brilliant results are seen when the operation is done within 24-36 hours from the beginning of the attack. After that time up to five days let things alone. If cases do not begin to improve in twenty-four hours from their inception, cut in at once, but if they are mild and begin to decline, let them alone as they will get well without operation and a subsequent operation is attended with less danger to the patient.

Many rare lesions simulating acute appendicitis were mentioned, such as ulcer of the stomach, sprains of the psoas and iliacus muscles, rheumatism of the same or of the abdominal muscles, etc., and the advisability of making a very careful diagnosis discussed. In recurrent cases operate between the attacks as well as in all mild cases that suddenly become severe. In speaking of the symptoms, first the pulse was mentioned. An accelerated pulse is a constant symptom, usually over or near 100. Pain—an early sign, local, and significant of peritoneal invasion. Tumor—a sign of value, but frequently difficult to detect. Temperature—unreliable as a guide, but a gradual rise means a severe case. Nausea and vomiting—if persistent after twenty-four hours, is very significant of peritoneal infection. Distension of the belly—after twenty-four hours this is very unfavorable. Leucocytosis—this is the most valuable sign we have, for a rapid raise means a great deal and means pus, while a decrease means a cessation of the irritation and a subsequent recovery. Constipation—present usually, and tenderness, rigidity, and tympanitis are the remaining symptoms to look for.

Dr. Dennis then gave a detailed report of eighteen cases he had operated on within the last few weeks.

The cure of

MALIGNANT DISEASE BY SURGICAL INTERVENTION

furnished the subject of the closing remarks of Dr. Dennis, but he requested the secretary not to publish them as he wished to record a series of 100 cases before submitting his views for publication.

A vote of thanks was unanimously tendered the distinguished visitor for the instructive evening he had afforded the section.

---

*Section of Ophthalmology, Otology, Rhinology and Laryngology, February 12, 1900.*

REPORTED BY RICHARD H. SATTERLEE, M. D., Secretary.

THE meeting was called to order at 9 p. m., Dr. W. S. RENNER in the chair.

Dr. Geo. F. COTT presented a paper on The Radical Operation, as applied to Middle-Ear Diseases (see p. 666).

DISCUSSION.

Dr. LUCIEN HOWE.—As to the anatomical relation, Dr. Cott did not mention the difference, as many cases of this nature occur in childhood. I appreciate the difficulties specially when operating on children. The operation in children from the proximity of the facial nerve makes the danger of facial paralysis very great.

Dr. E. E. BLAAUW.—I think we must congratulate Dr. Cott on his fine results. I think the success in getting union by first intention is due to the climate of this country. In the old country it is most difficult to get union by first intention after such an operation. Here the ear is pushed back and heals readily, while over there the difficulty is with an edematous mass which occurs usually after operating. In the old country these cases are kept in hospitals for months after, while here the case leaves the hospital usually in four days. About the hearing; why they hear so much better is because the whole bone conduction is better.

Dr. COTT.—I had to limit the length of the paper, hence I did not go into the anatomy as Dr. Howe suggested. In the very young child there is practically nothing but the tympanic ring which makes the operation more difficult, as Dr. Howe also points out. Facial paralysis never lasts if the nerve has not been injured. Sometimes in healing, granulation tissue will press upon the nerve, producing paralysis, but this is only temporary. Regarding such prolonged treatment in the hospital, I think this is unnecessary. I only take cases to a hospital at all, merely because it is more convenient to operate there. I do not mean by this that I dismiss the cases when they leave the hospital; on the contrary, they are under observation for months after, to see that no complication arises. The theory about hearing has not been satisfactorily settled. There are many things about the ear concerning which we are as yet in ignorance, but the day is surely coming when ear surgery will be done as understandingly and with as good results as abdominal surgery.

---

*Section on Medicine, February 13, 1900.*

REPORTED BY J. C. CLEMESHA, M. D., Secretary.

THE meeting was called to order at 8.40 p. m. by Dr. E. H. LONG, Chairman.

In the absence of the regular secretary, Dr. DEWITT SHERMAN was elected secretary *pro tempore*.

Dr. B. G. LONG made a report concerning measles in a family in which during an attack the mother was confined and the baby was born with a typical measles eruption.

Dr. A. A. JONES reported a patient who had typical measles for the second time, showing Koplik's spots during the second attack.

Dr. DELANCEY ROCHESTER reported a case of convalescing scarlet fever which had a second attack of the disease followed by a serious nephritis.

Dr. CHARLES G. STOCKTON took exception to the statement, that recurrences of measles were common; in his belief and experience such recurrences are very uncommon indeed.

Dr. E. H. LONG reported a patient suffering from scarlet fever with a very high temperature, decided angina, cyanosis, etc., with desperate symptoms. Antistreptococcus serum was used at two hour intervals, in all, 40 c.c. Later, the temperature again rose, when abscesses formed in the neck, which were incised and four ounces of pus released.

Dr. M. D. MANN reported the findings of a committee appointed by the American Gynecological Society on the subject of antistreptococcus serum. The conclusion of the committee was that this serum was nearly valueless, or at any rate its action was very uncertain.

The paper of the evening was then read by Dr. Mann, whose subject was

DRESS REFORM.

He said the follies of fashion have been for so long and are still so connected with women's attire that it is hard to separate the contempt they have engendered from its proper consideration. There seems to be innate in the human mind, soon after it emerges from the state of lowest barbarism, a tendency to modify natural forms, as witnessed by the Indian woman of Alaska, who puts a pin through her upper lip causing it to fall over the lower one; the women of China, who squeeze their feet to a shapeless mass; and so it is in various people throughout the entire world, each have their special mode of causing an artificial effect to their forms or features.

Civilised women endure a waist disproportionately small and commence at the early age of childhood to attain the desired effect. In this way they deviate from the well known classic forms which have been and are the standard of beauty. Taking the Venus de Milo as the highest type, we find the proportion of waist to height is as 47.6 to 100. In other words, a well developed woman to be properly proportioned according to the classical standard should measure round her waist nearly half her height; for example, a woman 5 feet 6 inches tall should have a waist measure of about thirty-one inches.

Measurements made from a large number of American women who wear corsets show a ratio of waist to height of 39.6. Similar measurements of Chinese, Japanese and Indian women, who do not constrict their waists, correspond to the classical standard, and some even exceed it. The conclusion is that the small waist is unnatural, inartistic and opposed to all that the best taste demands for beauty.

The corset does not come in for all the blame in the development of the narrow waist. The ordinary dress consists of three garments hanging from the shoulders, six from the waist, so that we find usually, counting each band as two thicknesses, sixteen thicknesses around the waist. Such an arrangement put on a growing girl cannot help but result in a greatly constricted waist.

From the physician's point of view the constricted waist is a deformity, giving rise to many displacements of abdominal organs, stomach, large intestine, liver, kidneys and certain more delicate organs below. In a large number of men and women examined, it was found that visceral prolapse was nearly seven times as frequent in women as in men. Among other evil effects caused by corsets and tight clothing, is an abnormal respiration, weakened abdominal muscles, protuberant abdomen, with a local accumulation of fat, and curvature of the spine, either lateral or posterior.

Now as to the proper hygienic dress, which requires four garments: First, a union undergarment; second, in cold weather, equestrian tights; third, muslin waist and skirt in one piece; fourth, a dress so made that its principal weight will fall upon the shoulders and be distributed over the bust and hips. No corset, no corset waist, no bands around the waist, no belt, everything free and flowing. It is admitted, however, to obtain this ideal "a campaign of education" is needed. It is only by education on what true beauty really consists, on a spreading of a knowledge of the rules of art, and a study of and familiarity with classic models that we shall come to realise beauty of form and grace of vesture in daily life.

DISCUSSION.

Dr. H. R. Hopkins said that the principles of reform in this direction should be that of beauty, rather than the hygiene of dress as ordinarily understood. He said that there is no one cause of bad development in women, for the Cadets at West Point show the same disposition as described by Dr. Mann. Proper training and development correct this. Referring to the statement that there is no waist-line in nature, he invited attention to the wasp and greyhound as examples of narrow-waisted insects and animals.

Dr. B. Bartow stated that the proper athletic training in young girls would overcome the necessity of dress reform, because after becoming accustomed to free and unimpeded body movements tight dresses would not be tolerated. He affirmed that the use of the bicycle has done more than any one single circumstance to advance dress reform.

Dr. DeLancey Rochester concurred with Dr. Mann in the opinion that dress is the chief cause of deformity in women, and stated that the figures presented by the essayist were very convincing. He suggested the introduction into the public schools of proper physical culture. This would necessitate reform in the present fashions of dress, as the girls could not exercise under its restraint.

Madame Mankell stated that corsets are probably the most harmful part of women's present dress. She advised half measures at first, after which more radical means could be insisted upon. The costume which she approves would allow free expansion of lower as well as upper chest. If corsets are removed training becomes necessary so as to avoid looking slouchy.

Dr. A. L. Benedict said that no standard for women could be applied as rigidly as for men, as many women cannot stand the weight of their clothing from the shoulders.

The meeting adjourned at 10.45 p.m. Twenty-three present.

